# Positive Influences: How Provider Actions Affect HIV Care Engagement for Black Women in the Southwest U.S.

**DOI:** 10.3390/ijerph22091319

**Published:** 2025-08-25

**Authors:** Kenja S. Hassan, David W. Coon, Johannah Uriri-Glover, Marianne McCarthy

**Affiliations:** Edson College of Nursing and Health Innovation, Arizona State University, Phoenix, AZ 85004, USA; david.w.coon@asu.edu (D.W.C.); johannah.uriri-glover@asu.edu (J.U.-G.); mccarthy@asu.edu (M.M.)

**Keywords:** HIV, patient–provider relationships, retention in care, care engagement, adherence, African American/Black women, positive affect

## Abstract

Despite medical advances having made HIV a survivable condition, HIV persists as the 11th leading cause of death among young Black women. Enhancing the quality of care engagement through beneficial patient–provider relationships can close gaps in retention and adherence, enabling long, healthy lives. Using constructivist grounded theory informed by an established framework for patient-centered care in complex cancer settings and insight from local HIV advocates, this work identifies what provider actions retain women in care and why. Through focus groups and interviews, eleven Black women in the Southwestern United States, an understudied population, express that providers who engage them as co-creators in maintaining good health are more likely to retain them. Concurrently, when women are attuned to their own health care and interpersonal needs, they discern which providers are equally committed to their health based upon observed provider actions. These actions, such as listening attentively, taking time, and paying attention to the whole person, in conjunction with women’s motivation and active involvement, create a reciprocal dynamic that increases the likelihood these women will remain virally suppressed. The ideal relationship is one in which the provider empowers and champions women as drivers of their own care.

## 1. Introduction

African American/Black women continue to bear the highest HIV burden among all women in the United States. HIV is the 12th leading cause of death for Black women ages 25–44, according to recent national mortality data [1]. Despite medical advancements that have made HIV a treatable condition, Black women are over 13 times more likely to die of HIV than white women [2]. Retention in care is still a challenge for African Americans nationally, with 52% retained in care and 61% virally suppressed [3]. Meanwhile, it has been long established that closing gaps in retention is linked to reduced mortality rates [4,5], and people with HIV can live nearly as long as people without the virus if they are in consistent anti-retroviral therapy [6,7].

Qualitative and quantitative studies on HIV patient–provider relationships found that trust in providers increases the likelihood of retention in care and adherence to antiretroviral medication, especially for African Americans; conversely, mistrust can have the opposite effect [8,9,10,11,12,13,14,15]. Studies have also shown that relationships with providers are a facilitator to staying in care, especially when patients view their clinics as safe spaces that are free of stigma related to HIV, race, class and gender; when they feel a sense of emotional support from providers and clinic staff; and when clinicians understand the unique life circumstances that may pose barriers to care [16,17,18,19,20,21,22,23]. Women are particularly responsive to providers who demonstrate knowledge of them as individual persons, who treat them with a respect that recognizes their equality as human beings, and who view them without prejudice [24,25].

In response to these gaps in retention, the CDC exhorts clinicians to use supportive communication and other active engagement strategies as a means of encouraging adherence to care [26]. This study created an opportunity for HIV-positive women to offer their own explanations of provider actions that have been favorable to their retention in care, thereby adding emphasis and clarity to the CDC’s recommendations of engagement style.

## 2. Materials and Methods

Through a qualitative approach, this research study sought to ascertain which provider attributes and actions, inclusive of communication styles, contribute to successful retention in care from the perspectives of HIV-positive Black women and why. Constructivist Grounded Theory (CGT), as described by Kathy Charmaz [27,28] is used as the guide for this study. CGT is based on the notion that people construct their reality and sense of self through interactions with others, and thus CGT methodology acknowledges the complexity of social interactions [27,28]. Due to potential differences in race, education, socio-economic status, and perceptions of the medical system, patients and providers may hold very different assumptions about each other and how to achieve the best health outcomes. This method also encouraged an understanding that data and theories are constructed by the researchers as well as the participants. The process developed for this study purposely built in opportunities for multiple individuals to scaffold and inform these data: local HIV advocates, who were interviewed for context and recommendations on how to ask HIV patients questions; and HIV patients themselves, who offered input regarding the interview questions during focus groups, clarifications on their answers during interviews, and ideas to consider when discussing this information with other participants.

### 2.1. Sensitizing Concepts

Per Charmaz’s [28] recommendation that some research be guided by sensitizing concepts rather than a complete blank slate, Epstein and Street’s *Patient-Centered Communication in Cancer Care* [29] was identified as framework to organize interview questions. Ultimately, this framework proved to be a highly adaptive guide for understanding the relational needs of this population, albeit with considerations for HIV stigma, past mistrust of the medical system, and the skewed impact of both on low-SES patients.

Epstein and Street’s *Patient-Centered Communication in Cancer Care* (PCC) [29] provides a comprehensive, morally grounded framework for effective provider attributes and communication skills, rooted in valuing each patient’s unique life context ([29] p. 1). Epstein and Street’s [29] framework organizes six key communication functions that are equally relevant in the socially and medically complicated context of HIV care: (1) fostering healing relationships, (2) exchanging information, (3) responding to emotions, (4) managing uncertainty, (5) making decisions, and (6) enabling patient self- management. This framework for provider communication forms one-third of a three-way system that Epstein and Street [29] say leads to effective health outcomes. The other two components are a high-functioning health system and what they describe as an “activated” and engaged patient. Participant responses in this study would require the addition of a seventh section to Epstein and Street’s [29] framework: the patient contribution to the relationship. Participant responses revealed that true success happens when the patient herself is motivated, and true confidence in her care happens when she sees the clinic staff working as a team among themselves and with other providers.

### 2.2. Setting and Data Collection

This study occurred in a large metropolitan area in the Southwestern United States. As no previous studies have been published on this topic in this region, this research project involved a lengthy process of understanding the local context by interviewing HIV advocates familiar with both the population and services available before approaching potential study participants. Among the group of twenty-four HIV advocates sought for advice were medical doctors, case managers, HIV program administrators, non-profit employees, and organizational volunteers.

Each interview with HIV advocates, which lasted between 30 and 60 min, concluded with the advocate offering a list of questions that would help uncover important patient–provider relationship dynamics. These questions were then organized according to the six major themes identified in the PCC [29] as critical to patient-centered communication along with the seventh section on patient contributions. Most of the advocates recommended questions that dealt with how providers developed trust and rapport. These were placed in the category “fostering healing relationships,” which became the largest group of questions. Another area of emphasis was “responding to emotions” because of HIV stigma, stereotypes tied to Black women, and the unique challenges faced by Black women.

Black women living with HIV (WLWH) were recruited through flyers posted at local HIV clinics, AIDS service organizations (ASOs), and by referral from HIV advocates. Recruitment flyers described all participant criteria as well as privacy and consent information. The criteria included being over 21 years of age, having sufficient English language comprehension to understand the consent form, self-identifying as female and Black or African American, being HIV-positive for more than one year, and having a self-rated adherence of good or excellent regarding engagement in care.

Interested participants contacted the research team by phone and were screened, while their contact information and eligibility were logged on screening forms. During two focus groups, four participants who met the study criteria reviewed, offered edits, and approved the appropriateness and usefulness of the interview questions. Focus groups were held in a private conference room at an ASO familiar to participants, and lasted approximately two hours. Participants were provided with snacks and space for children to do homework. The final individual interview questions were open-ended, and follow-up questions were asked for clarification. Individual participants were interviewed at a location of their choice, most often their homes or a private office at a local university. These interviews lasted between 60- and 90-min. Consent was provided in-person prior to the focus group or interview. Participants received a USD 15 gift card for participation in focus groups and interviews, totaling USD 30 if they participated in both. 

#### Example Interview Questions

1. Think of the best provider you ever had in HIV care. How did that provider treat you?
2. Think about times when you have felt uncertain or fearful about your healthcare needs. What might make you fearful to have a conversation with your provider?
3. How much of your life aside from HIV do you share with your provider?
4. What do you think you bring to the relationship with your provider that gets you what you need?

### 2.3. Data Analysis

Interviews were digitally recorded and transcribed using an automated service. The transcripts were reviewed and verified by one researcher. Consistent with Charmaz’s [27] recommendations, data analysis was concurrent with data collection in that participants were asked to verify the researcher’s understanding of their comments during interviews or focus groups, and analysis occurred while transcripts were being verified. Charmaz’s [27,28] analytical guidance orients researchers toward the ongoing social processes involved in human interaction while acknowledging that there is no singular objective reality. For this study, it was important to be cognizant that gender, income, race, and educational differences would impact the social interactions of these women with their providers and that interactions might carry different meanings to different patients. Researchers were sensitive to these dynamics throughout data analyses.

All coding was performed manually. The lead researcher used line-by-line coding to generate initial themes. The initial themes were reviewed by the qualitative researchers in the team and discussed until all were in agreement. A codebook was then created and the lead researcher completed coding manually with periodic reviews by the other members of the team. Once all interviews were coded, themes were then compared among the interviews and coalesced into larger themes. The research team discussed analytic and theoretical memos to determine salient themes. Larger themes were compared to existing themes in the PCC [29] to determine their fit with this population.

### 2.4. Author Reflexivity

Charmaz [27,28] emphasizes that authors should be open about their intent and positionality because they are constructing data and theories rather than claiming or attempting to witness data objectively. Two members of the research team are Black women who believe that their positions as visible community members oblige them to advocate on behalf of Black women’s health. A third member of the team is a white man with experience as a psychologist working with people with HIV. The fourth member of the team is a white woman with clinical expertise in adult mental and behavioral health nursing. Combined, the researchers view this work as an opportunity to document steps that may help Black women achieve parity with other people with HIV. The researchers acknowledge that they have more formal education than most of the participants and are aware that this educational difference can influence how they interpret participant responses. The researcher conducting the interviews, herself a Black woman and non-clinician, sought to engage participants without conveying judgment, inadvertently using stigmatizing language, or conveying a notion of social hierarchy.

## 3. Results

### 3.1. Participants

Thirteen women met the criteria. Table 1, Table 2 and Table 3 summarize the number of participants in each stage of the study, their characteristics and their engagement in care. Two participated in the focus groups only, nine completed interviews, and two were not able to participate due to travel and life changes. All participants self-identified as Black and female. None identified as Hispanic, and none reported that they immigrated from any region outside of the United States. All of the women were over forty and had been in HIV care for at least ten years. Most of the women had children. The majority of participants self-rated their medication adherence and retention in care as good to excellent based on how frequently they took their medications and stayed on track with the CDC definition of retention as taking at least two tests (CD4 or viral load), at least three months apart, per year [30]. All had worked with HIV case managers for some period of time.

### 3.2. Predominant Themes

Most of the participant responses paralleled the communication strategies and provider actions recommended in Epstein and Street’s handbook on *Patient-Centered Communication in Cancer Care* [29], but with added recognition of the patient’s own role. Epstein and Street acknowledge that an “activated” cancer patient will have greater success than one who is disconnected. Meanwhile, the women in this study made it clear that the ability to combat HIV is seeded by self-motivation and fostered by a dedicated provider. Thus, two major areas emerged as fundamental to successful retention in care: provider actions that engender trust and women’s active participation in their own care.

#### 3.2.1. Provider Actions That Generate Trust

**Fostering healing relationships**. The lengthiest segment of all interviews was the section related to “fostering healing relationships.” Most of the advocate-recommended questions aligned with this concept and several women relished the opportunity to describe their best HIV provider. Participants identified the following actions as those that motivated them to return: listening, taking time, paying attention to other parts of the patient’s life, showing respect, relating and being relatable, being understanding and nonjudgmental, being thorough and diligent, being truthful and straightforward, making physical contact, working cooperatively with other providers, asking the patient questions and not assuming the patient knows what to tell the provider, being knowledgeable about HIV and potential resources, and taking action when a patient is in crisis.

**Listening.** Listening was cited nearly as often to describe a good provider relationship as its absence was to describe a bad provider relationship. Women said their best providers authentically heard the specifics of their situations. “They’re willing to listen, hear me and I mean really hear me, because some people don’t hear; ‘cause we’re all different and this disease is different in all of us,” one participant explained.

**Taking time.** A provider that takes time instills confidence: “I know that if I have an issue or whatever, that my provider will sit down and take time. She will take the time if I have an issue and we’ll discuss it. I don’t feel rushed,” said one woman. In contrast, rushing was the top issue that generated fear about talking openly with providers. When asked to explain further, the women in this study said that rushing made them anticipate that the provider would dismiss their concerns or input.

**Showing interest.** Participants indicated that good providers show an interest in a full understanding of the patient’s life. This made them feel important, and, they noted, helped the provider discern what other medical or life issues may impede retention in care. The interest should be communicated authentically: one participant stated that providers who sound too sweet or ”too sugary” remind her of a car salesman, hinting at untrustworthiness. Conversely, genuine attention and concern from the provider became internalized in the patient. As one woman described it, the attention and concern from her provider was similar to the feeling of having a teacher or coach who took pride in the success of those they help.

**Showing respect.** These participants described demonstrations of respect in many forms. This is evident when providers discuss disagreements rather than dismissing patients’ views that don’t align with theirs; when they demonstrate to other providers that they are committed to their patients’ well-being; and when they don’t “talk down” to patients. Being direct was valued, and being overly gruff or dictatorial was not: one woman described a provider speaking to her “like she was [her] mother.” This style came across as patronizing and not valuing the woman’s abilities. It is also evident when they make the patients feel like they are human beings, as one interviewee stated: “They did that by taking the time to see what was going on with me. They didn’t see a color or race or anything like that, they saw an individual.”

Closely tied to showing respect is not showing judgement. One woman said she could tell her provider everything and that kept her safe from self-harm: “If I couldn’t tell her those things, I would probably be in a little trouble because then I would be keeping it to myself and if I keep it to myself then I’m gonna do what I think I should do and that’ll be ending up using. And I don’t want to use again.” Women in this group did not want to be cast as promiscuous or drug abusers, and recognized that providers who work in HIV must understand the social taboos PLWH must navigate. One woman explained that providers should be aware that HIV is a “mentally painful disease,” sometimes more emotionally than physically painful.

**Relating.** Relating to the patient and being relatable themselves could be interpreted merely as having a good bedside manner and friendliness, but these women looked for something deeper. They looked for how the provider related to them as an indicator of whether or not the provider is in the job for the health of their patients or “to collect a paycheck” as one said. For some, this meant the providers sharing some information about themselves. For two, this included an apparent awareness of the African American historical context and its impact on health.

**Investigating diligently.** Women were impressed and comforted by providers who were thorough and diligent. Such providers were willing to order more tests, do more research, look deeper into the women’s health challenges, and explore medicines for them. The hallmarks of a thorough provider were ensuring that all of the tests are up to date, having knowledge of the patient’s specialists, asking about the side effects of medications, and familiarity with the patient’s chart before she comes into the room.

**Working as part of a team.** Women were attentive to how well their providers work with other providers and identified teamwork as a critical attribute. Participants noticed if providers were willing to give referrals and if they called other providers when situations were uncertain. They were impressed and felt confident when providers inside a clinical setting worked well together, but especially when they worked well across systems.

**Understanding HIV.** These women valued providers who were knowledgeable about the basics of HIV and were willing to keep up with new developments, such as new medications and U = U (Undetectable = Untransmittable), the recent data that shows people who have an undetectable viral load cannot transmit the virus, as per the CDC [31]). Giving thorough explanations about the virus helped people feel comfortable, and they knew enough to know the difference. As one woman explained, “I’ve only been to two who really know and keep on top of their information.” She, like almost all of the participants, saw an HIV specialist.

**Responding to emotions.** In tense situations, how a provider responds to emotions while under pressure is especially important to maintaining a productive relationship. These women identified several ways in which a provider can calm a situation gone awry. They can ease women’s fear by talking with them and providing information, or they can request the assistance of social workers, but they must show concern, take action, and be available to answer questions or have staff who can do so. One woman explained that her provider approached her with compassion when she was depressed. Instead of brushing off her depression, “He sat down with me and talked to me and calmed my fears.” About half of the women are able to describe crisis situations when providers did not take action, and compared that experience to one with a provider who did.

#### 3.2.2. Inherent Provider Attributes

In general, inherent attributes (race and gender) were less important than how the provider behaved. Most participants said that they did not have a preference regarding the race or gender of the provider, so long as they were treated with dignity and respect. While many indicated they would see a Black provider if one were available, overall, how the provider made a connection with them was more important than their race. Those who preferred female providers said this was because of their ability to relate to them emotionally and have a deeper understanding of their challenges. When describing their “best” provider, seven indicated that person was female and four indicated male, either outright or through the pronouns they used.

#### 3.2.3. Types of Providers

The women were encouraged to describe their “best” HIV provider and the interviewer did not probe for professions or names, only traits and actions. Nevertheless, five women excitedly described providers with whom they were enamored. Two of these were physicians and one was unclear. One woman’s top choice was a case manager and another woman’s top choice was a doctor in nursing practice.

#### 3.2.4. Patient Attributes That Contribute to Good Health

**Relationship with self.** Women remarked that an HIV-positive person must “go through an internal process,” in order to “come to grips” with their diagnosis. Several described needing to get over denial or depression. These women recognize that this is a serious illness and their desire to be healthy overrides any fear of stigma or other challenges that might arise. One participant cautioned, “Staying in denial will kill.”

Having self-assurance increases the effectiveness of relationships with providers. This self-assurance may come from religious views, spirituality, or a deep sense of self-respect. Women often said that what matters most in managing HIV is how they take care of themselves and what they think about themselves, as this woman illustrates, “It’s up to you, it’s how you care for yourself, and how you treat yourself, and how you love yourself.” This is not easy or automatic. They say that they have to encourage themselves and not waste time on self-pity. Some find consolation in their church, with a pastor, or a faith community. Some see that having this virus gives them meaning and purpose to learn how to face challenges and then offer guidance and assurance to others.

Knowing that they are more in control of their lives gives them power over the virus, “I realized that my attitude was paramount in this disease, my own self-attitude,” one said. Another explicitly acknowledges her power: “This disease is not in control of me. As long as I know I have the power over this disease that gives me the leverage to do whatever I need to do.”

Most of the women stated clearly that they “knew” their bodies and that physical awareness was an important part of their self-assurance when working with providers. Being very familiar with one’s body and how it feels when it is healthy enables the women to know when something is very wrong. Four of the women had experiences with life-threatening or excruciating symptoms that providers dismissed. In each case, the women pressed ahead to find a provider who believed them. In addition to their physical health, many also realized that they had to pay close attention to their mental and emotional health.

Understanding the dangers of HIV motivated some women to stay in care. Some of them had seen others suffer and die from HIV-related complications. They know that their lives are under threat if they are not virally suppressed, as one woman points out, “something simple like the common cold could kill you.” They are committed to regular testing and understanding their target numbers. Keeping up with new information and being educated about the virus gives strength, “When I’m educated, I’m empowered,” one explained.

**Purposeful interaction with providers.** While women recognize that they are in control of their health, they also know that they would eventually die from complications due to this virus without professional help. Therefore, they are conscientious about their interactions with providers. Moreover, women in this study recognized that the providers are motivated to help them more when they can show their provider that they are following through on recommendations. Two women explained that providers can see that they are serious about their care when they are organized and consistent, for example, “Coming in with questions and being prepared lets the provider know you are taking charge of the virus and your health.”

“When you raise the bar, they raise the bar,” one interviewee said. Many of the participants prepare in advance for appointments with providers. Some of them have kept notebooks, one uses the MyChart app (she did not specify which version) another writes down what she hears from the provider during the appointment and then repeats what she heard back to the provider in order for them to clarify her interpretation of their words. Another brings a copy of the lab work with her every time and does not leave the appointment until she understands the readings.

Being able to ask questions of the provider is very important, these women say, because not all providers offer information. The women indicated that they know to ask about side effects regarding medications, clarity on test results, and non-medical resources.

The majority recognized that they could influence the quality of their care engagement by setting the stage for an upbeat and productive conversation with their provider, even when that provider seemed not to be at their best. Many were consciously aware that if they approached providers as if they were people who wanted to have a good workday, they could also have a good appointment. One said she talks with them about normal things like their families and their kids. Another was strategic about her role in creating the kind of partnership she wanted to have with the provider, in that she planned health-related questions and set aside stressors before entering the exam room so she could be fully engaged with the provider. Another recommends that patients start with small things and become more engaged as trust is built. Others say they just walk in with a smile no matter what else is happening.

**Discussing problems.** These women did not shy away from medical problems. They were ready to talk with their providers, especially as it concerns medication. They were committed to keeping their appointments even when they did not want to, or did not like, going to the doctor. These participants stressed that being honest with providers is essential to receiving the level of attention they need. One emphasized that “if you provide good information, they can provide good care.” Those who have substance addiction were able to explain when and why they were using substances to their providers, which they believe can help the provider get to the root of a particular challenge.

**Seeking social support.** Women frequently mentioned reliance on other individuals and groups for social support, emphasizing its importance to their quality of their lives. These women recognized it was important to not isolate themselves, including those who are not open about their HIV status. Two of the women spoke highly of other HIV-positive women who taught them about self-care and securing resources. Social support among these interviewees included family, church members, friends, and institutional support, such as employees of AIDS service organizations, support groups, and patient navigators. Some of the women had brought friends or family to appointments when they needed someone to take notes, ask questions they might forget, and when they were dealing with a provider they were not sure about. One spoke of how women from her support group had to go to appointments with a woman who experienced debilitating side effects from medication but was too afraid to talk to her doctor.

**Deciding when to leave a provider.** Having the fortitude to leave a provider, so long as their insurance allows it, gave these women greater control over their ability to find a provider with whom they can create a partnership. Women in this group advocated for themselves, arguing “It’s OK to stand tall, to stand firm on your life…If it’s not working for you, move on to the next.” One advises patients to pay attention to the provider, and specifically to ask oneself, “does he or she take an interest in who you are? Do they take the time to listen, really hear you out?”

When a relationship has gone bad and they cannot leave, they have tried to re-approach providers, but with mixed success. Two have simply stayed with providers out of need, but make it a transactional relationship. This may not be the best outcome for HIV patients who have less stable lives.

## 4. Discussion

The purpose of this study was to learn what patient–provider interactions are successful in keeping HIV-positive women in care and why are they effective by using a grounded theory qualitative approach guided by *Patient-Centered Communication in Cancer Care* [29]. These participants identified actions such as listening, taking time, and showing respect, which have been identified in the PCC framework and by researchers who established that patient-provider relationships influenced engagement in HIV care [17,18,23]. The facile answer as to *why* such actions matter is that they make these women feel like valued human beings. Feeling valued is critical for any patient population, yet this is a group that has been historically undervalued, which in turn can make them skeptical and even suspicious of the medical establishment. Furthermore, when the nuances of how actions impact women are explored, providers can have a stronger sense of how their behavior affects patients’ engagement and why they should emphasize some actions or behaviors and avoid others.

While the women who participated in this study were engaged in care and willing to discuss HIV openly, they were also susceptible to the kinds of life circumstances that create barriers to care and that could lead to poor retention, emphasizing the need for patient-centered care. Without being prompted on their personal lives, women described life situations that reveal the kind of instability that can put their retention in care at risk: incarceration, intimate partner violence or emotional abuse, unstable housing, difficult medical conditions, fluctuating employment, child-care and other care-giving challenges, lack of reliable transportation, financial poverty, emotional and psychological distress, substance abuse, care access through public health coverage as opposed to private insurance, and more, all of which came up in the course of interviews. Each revelation either gave greater context as to why a particular provider had an impact on them or illustrated their personal resilience. These life circumstances accentuate why the relationship to oneself and the relationship with the provider become amplified in their HIV care engagement.

The importance of taking time was accentuated when women discussed how rushing made them feel. In other studies, women expressed that they preferred providers who did not rush through the appointment [32], but what bothered them about rushing was not described. In this study, women explained that a rushed provider made them feel less important, more fearful of speaking up, and possibly forget what information they needed to ask about or convey.

Several women described a preference for genuineness in communication. Being friendly but not “phony” was a trait that was also found to be important for HIV-positive women elsewhere [21]. When probed why too much friendliness was problematic, women said providers who “sugar-coated” information, or who tried to sound overly sweet in their delivery or too timid in their directions, appeared untrustworthy.

Certain actions show women that a provider is committed beyond the paycheck. Teamwork, working cooperatively with other providers, and being willing to make referrals indicates that providers know the limits of their skills, rather than attempting to be the authoritative figure at the expense of the woman’s health. This means they may also be willing to dig deeper and ask questions on behalf of their patients. Being thorough and diligent gave women confidence that providers were interested in their unique situations and were prepared to go to lengths for them.

Being relatable and non-judgmental carries additional weight with these women because of the stigma associated with HIV, but these traits are also medically important. As McDoom et al. [33] found, women’s perceptions of what the doctor thought about them influenced their interests in staying with that provider. In agreement with this finding, participants said they had providers at some point who “looked down on them,” which was detrimental to their motivations. Deepening McDoom’s [33] findings, these women pointed out that not feeling judged gave them a sense that they could ask questions and be open with providers about problems that could affect their health and even prevent self-harm or self-medicating through alcohol use, substance abuse, or other forms of addictive behavior. 

Providers who update women on their health progress in an encouraging manner provide a unique type of motivation and social support that women could not get elsewhere, especially if they were not open about their status or had isolated lives: “I was not only dealing with HIV but I was dealing with an abusive husband… So, the encouragement of providers and the support groups were really important.” Having a supportive provider is like having a cheerleader who understands the unique challenges of their life and health goals: “It’s really encouraging to have someone that you can be yourself with and know about your situation and not bring you down and be negative about it, but they’re there to uplift you and tell you to keep up what you’re doing.”

Paying attention to other areas of the woman’s life beyond HIV and listening closely to the woman are both motivating to her and are medically practical. One participant who volunteered to help women with HIV explained that many of the issues that keep women out of care are not related to the virus itself; therefore, providers who ask about the woman’s whole life can be better prepared to discern what help she needs to stay in care. The women who had comorbidities felt like HIV was the easier illness to manage once they got their medication on track. Those who knew they had complicated medical histories needed providers who were prepared to help them navigate more than just HIV. One woman who suffers from depression and substance abuse reported that it was very motivating when her new provider asked about her anti-depressant medications and support needs. This showed her that the provider was concerned and that managing her depression could prevent substance abuse and help her stay in care. Careful listening creates a strong relationship dynamic and demonstrates to the woman that the provider is seeking to understand her individual situation.

In the course of describing their best provider, several mentioned that providers sat on their bed, held their hand, and gave them hugs. For this stigmatizing illness, touch is a demonstration of human kindness, but also shows the providers’ full confidence in how the virus is transmitted. One of the women told medical students studying to be HIV specialists that they should be willing to hug their patients in order to build trust. As is the case with Black women elsewhere, interactions with providers are an important source of social support, especially for older patients and for those who may experience intrapersonal or interpersonal stigma [23,33].

All of the women said it was important or very important that they be involved in decisions regarding their health care. Women in this study indicated a willingness to seek and utilize resources available to PLWH, such as working with HIV navigators early after diagnosis. Marelich and Murphy’s [34] quantitative study on patient empowerment found that patients who were involved in the decision-making process also had greater communication with their providers. Through the use of qualitative methods, the present study revealed that providers who set the stage for women to be involved in their health decisions through actions that generate trust assures the women that the providers see her as the driver of her health. This provider-supported empowerment reinforces the patient’s commitment.

All of these actions lend to the creation of a partnership, which seemed to be the ideal provider relationship for these women. Okoro and Odedina (2016) [19] interviewed both patients and providers on facilitators to HIV care. The providers in their study understood that they needed to figure out how to create a partnership with their patients. This desire for a partnership holds true for Black women in opposite sides of the country. A next step following this study would be to interview the HIV advocates on what actions they perceive help build a partnership and compare those to what women have said.

Importantly, these actions all contribute to the woman’s ability to grapple with HIV, become comfortable with medical knowledge, and build confidence with self-care. As noted, most of the participants said it was important for any newly diagnosed person to come to terms with their diagnosis, move past denial, overcome stigma, manage external barriers, and take responsibility for their own health care before they can be successful working with any provider. Providers who can engender self-care and life management are logically the ones these women seek.

### 4.1. Application of the PCC Framework

Patients living with HIV, like many in this study, can have a degree of personal or medical complexity that requires attention on par with cancer treatment settings. Women explained that sharing their knowledge about their own bodies and symptoms with providers was important to knowing both parties understood their unique experience with HIV, comorbidities, and medication side effects. All of the six areas of the PCC framework resonated with the communication strategies and actions that participants in this study indicate as effective, especially the methods for developing trust and rapport. The five other parts of the framework are also highly instructive for this population: exchanging information, responding to emotions, managing uncertainty, making decisions, and enabling patient self-management. A strong example of this applicability is Epstein and Street’s [29] endorsement of a “process model of information exchange” ([29] p. 21) rather than a one-way transfer of information from provider to patient. They advocate a “reciprocal effort” between both parties to come to “a shared understanding of the medical and personal issues underlying the patient’s health condition” ([29] p. 21). In addition to this shared decision-making model, the PCC model would advise that providers address women’s emotional concerns rather than dismiss or downplay them; seek information about women’s lives outside of HIV to identify potential derailers; and work with them on communication or information sharing between appointments, including with other providers so as to avoid conflicting prescriptions or instructions.

#### Additions to the PCC Framework for Black Women in HIV Care

The PCC [29] allows for flexibility, reminding providers to be prepared for cultural and linguistic differences. For this population, our findings suggest two additions to this framework calling upon providers to learn how stigma and historical trauma impact women’s psychological well-being and care-seeking behaviors. Stigma, discrimination, and negative perceptions tied to HIV acquisition are all barriers to care in the study region, as explained by the HIV advocates. The women living with HIV were adamant that they made a conscious choice soon after diagnosis that they were worthy of care, and this seems to be their antidote to stigma. HIV advocates also explained that women who made this choice were the ones most likely to be actively engaged in care. The women living with HIV described stigmatizing situations, such as providers wearing protective gear or assuming they were promiscuous, and that they would strive to find other providers instead of internalizing those actions. An expansion of the PCC would incorporate instruction on how stigmatizing behavior changes patient engagement.

The second proposed addition to the PCC [29] would also help providers recognize how historical trauma and current barriers to equitable care impact Black women’s trust in providers and increase skepticism that their understandings of their symptoms will be taken credibly. Moreover, some women may need emotional support with HIV in a way that is not typical with other illnesses, as social stigma can reduce empathy, even from family. Many women may need to hide their serostatus from others. In addition to the PCC’s [29] communication strategies, reflections on implicit bias and cultural differences in self-expression would be helpful for all staff working in HIV clinics, including front desk personnel.

### 4.2. Limitations

This sample size is smaller than other studies looking at HIV-positive Black women. Those studies, however, are in areas with large African American populations [18,19,20,25,35,36]. Despite heavy recruitment efforts among HIV advocates, few women were willing to participate, likely due to fatigue about discussing HIV overall or a desire to remain anonymous in a very small community according to some of the advocates. One participant expressed pleasant surprise when realizing the questions were not about her HIV problems, which she said she was no longer interested in discussing. No one with less than 10 years of HIV diagnosis completed interviews. Although women reflected on what would have made it easier for them when they were newly diagnosed, only one could offer experience in the current care environment for newly diagnosed women. This woman described her granddaughter’s experience as bleak compared with her own some 15 years prior in the same city.

Participants were recruited through referral, self-selection, and snowball sampling. Self-selection will skew these data toward women who are more confident and open about their HIV status, while referral and snowball sampling will skew these data to women who are linked into social support networks. This does not give us insight into women who are resistant to care or those without HIV-related formal support networks.

The interviews did not discuss ART initiation; however, all of the women alluded to the importance of prioritizing one’s health immediately upon diagnosis, regardless of all other circumstances, including external sources of shame or guilt. The HIV advocates also said that the women who are most likely to be successful at maintaining their health were the ones who were persistent about seeking resources right away. An opportunity for future research lies in how diagnosing providers can be trained to ignite something in the patient to make her feel worthy enough to preserve her life and engage in care.

Lastly, although over twenty HIV advocates were interviewed prior to approaching study participants for regional context related to HIV care engagement, their data were not explicitly analyzed for juxtaposition against the women living with HIV. This is an opportunity for future research.

### 4.3. Strengths

The method for generating interview questions was a collective process involving seasoned researchers and practitioners in the HIV sphere. The questions were edited and approved by participants in the focus groups who felt confident enough in the research to follow up with an individual interview. The participant responses aligned so closely with the principles described in the PCC [29] that they endorse fundamental provider actions necessary to build patient trust.

As noted above, studies in other parts of the United States demonstrate that relationships with providers are a facilitator to staying in care [17,18,19,21,22,23]. This study is both distinct from and complementary to this earlier research. This study is distinct in that it built on previous findings that provider relationships were essential to adherence and a facilitator to care, then framed questions around discerning which actions women thought were pivotal and how those actions either lent to their motivation or served as de-motivators. This study is complementary to this existing literature in that the participant responses in the Southwest aligned with responses given by women in qualitative studies in other regions around the country. This project added validity to previous findings and indicated some consistency among Black women, regardless of where they reside [17,19,32].

Within the context of the Southwestern U.S., where Black populations are small and yet make up a disproportionate amount of HIV diagnoses and mortality, a study centered on Black women fills a noticeable gap. Historically, the Arizona Department of Health Services has noted that the impact of HIV disparities is alarming among Black women in Arizona, but this group has not been the focus of published works, in general, and on successful patient-provider retention strategies in particular. In Arizona, Black populations are so widely dispersed that it is currently impossible for people to find geographic hubs with the kinds of social support that exist in urban centers with large Black populations; therefore, provider interactions become even more significant. This research can be instructive to providers in Western and Southwestern metropolitan areas with similar population distributions.

## 5. Conclusions

In conclusion, the focus groups and interviews revealed that women are most responsive to provider actions that demonstrate authentic connection, invested interest, and clear attention to them as individuals. Actions such as those identified in the results are effective because they create a dynamic relationship between the provider and the patient that builds upon the woman’s capabilities, capacity, and self-worth. Participants reported they are more likely to stay on medication, make healthy choices, and return for appointments when they know their provider pays attention to them, shows concern for their health, and demonstrates a sense of mutual accomplishment when the woman’s health is maintained or improved.

The women in this study indicated that self-motivation and/or motivation from children, friends, or other family was fundamental. What they expressed implied that women who do not have strong social support could be retained in care by a provider who ignites self-motivation and becomes a champion in the woman’s health care journey.

This study was unique among the HIV literature in that it used a framework for patient communication based on cancer care settings, yet found that the patient’s perspective matches that framework’s description of ideal patient–provider interactions. The overall alignment between these participant’s responses and the descriptions offered in the PCC indicates that Epstein and Street’s [29] work highlights strategies that are effective for a greater range of patient populations. Using the work of qualitative studies focused on people with HIV, an adaptation of Epstein and Street’s work could be included as orientation material for providers new to HIV care.

Further research should examine current provider views on how their efforts match the perspectives of women described herein. This research can lend to an understanding of how HIV care providers across disciplines can be trained in communication and relationship-building skills based on the PCC [29] framework.

On the whole, participants in this study are observant and astute when it comes to their health care providers despite coming from socio-economic strata that could lead providers to believe they are naïve about the medical system or how their bodies function. These women determine if a provider is a good match for them at an early stage of engagement. If insurance and availability allow for switching providers, most of these women would give a provider one or two chances before deciding to move on. They look for actions that show that the provider can view them as someone to be championed, not a reason to collect a paycheck. One woman captured the essence of this view: “Some people, you know that they are there on your behalf… and it’s not just a job, they want to see you do better, feel better, and to be victorious in all areas of your life”.

## Figures and Tables

**Table 1:** Qualitative design.

Format	Number of Participants
Two focus groups	4
Individual interviews	9
Combined total	11 *

* Two focus group participants did not interview.

**Table 2:** Participant characteristics.

**Race and Ethnicity**	
Black	11
Hispanic or Latina	0
**Age in years**	
Range	40–69
Mean	55.7
**Gender identity**	
Female	11
**Highest level of education**	
Middle school	1
High school or GED	2
Some college	3
Associates degree	4
Graduate or professional degree	1
**Number of participants with children**	**8**

**Table 3:** Engagement in care.

Provider visits per year (avg)	2.4
Self-rated adherence on a scale of 1 (poor) to 6 (excellent)	5.2
Number who have worked with an HIV case manager	11
Range in years with case manager	1–16
Mean years with case manager	7.5

## Data Availability

The data presented in this study are available on request from the corresponding author due to privacy concerns.
